# Supplementary material for: Identification of photoperiod-induced specific miRNAs in the adrenal glands of Sunite sheep (Ovis aries)
Source: Front Vet Sci. 2022 Jul 22;9:888207. doi: 10.3389/fvets.2022.888207 (PMC9354845; doi:10.3389/fvets.2022.888207)
Supplement: Supplementary file 1 [file Data_Sheet_1.ZIP › Supplementary file/miRDeep2 file.zip!/miRDeep2 file/Know_Structure/LP42A2/oar-mir-1185.pdf]

miRBase precursor : oar-mir-1185
Total read count : 211
oar-miR-1185-5p read count : 66
oar-miR-1185-3p read count : 145
remaining reads : 0

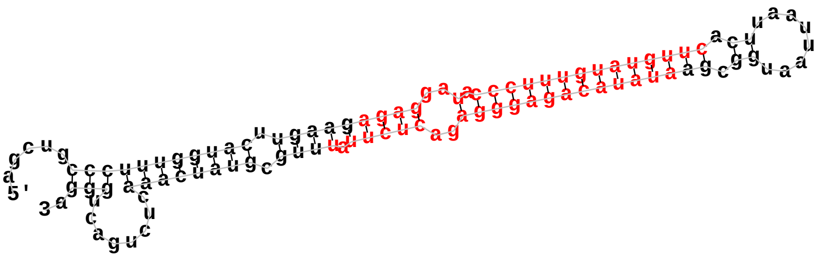

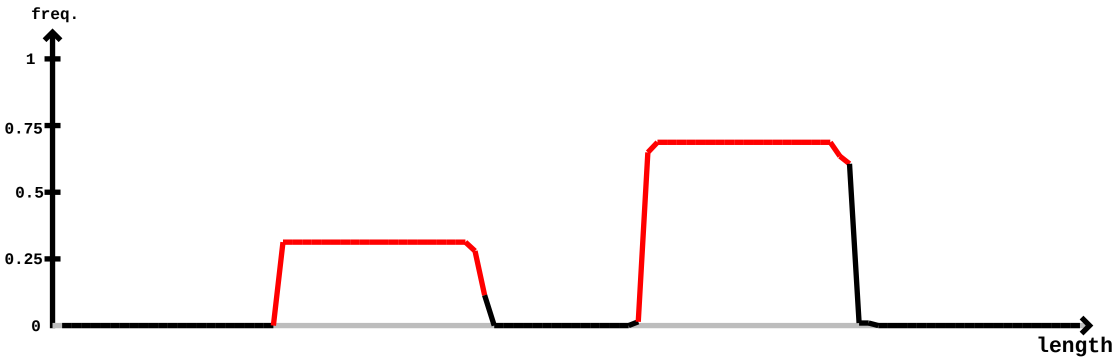


```
                     oar-miR-1185-5p
                                                        oar-miR-1185-3p
5'- agcugcccuuugguacuugaagagaggauacccuuuguauguucacuuaauuaauggcgaauauacagagggagacucuuauuugcguaucaaacucugacuggga   -3'    exp
    .....(((((((((((.(((((((((..(.((((((((((((((.((........)).)))))))))))))))..))))).)))).))))))))........))).    reads      mm          sample
    ......................agaggauacccuuuguaugu................................................................    7          0           seq
    ......................agaggauacccuuuguauguu...............................................................    35         0           seq
    ......................agaggauacccuuuguauguuc..............................................................    24         0           seq
    ...........................................................aauauacagagggagacucuuau........................    3          0           seq
    ............................................................auauacagagggagacucuu..........................    11         0           seq
    ............................................................auauacagagggagacucuua.........................    6          0           seq
    ............................................................auauacagagggagacucuuau........................    115        0           seq
    ............................................................auauacagagggagacucuuauuu......................    2          0           seq
    .............................................................uauacagagggagacucuuau........................    8          0           seq
```
